# Defunctioning loop ileostomy in anterior resection for rectal cancer and subsequent renal failure: nationwide population-based study

**DOI:** 10.1093/bjsopen/zrad010

**Published:** 2023-05-10

**Authors:** Martin Rutegård, Jenny Häggström, Erik Back, Klas Holmgren, Jonas Wixner, Jörgen Rutegård, Peter Matthiessen, Olle Sjöström

**Affiliations:** Department of Surgical and Perioperative Sciences, Surgery, Umeå University, Umeå, Sweden; Wallenberg Centre for Molecular Medicine, Umeå University, Umeå, Sweden; Department of Statistics, Umeå School of Business, Economics and Statistics, Umeå University, Umeå, Sweden; Department of Surgical and Perioperative Sciences, Surgery, Umeå University, Umeå, Sweden; Department of Surgical and Perioperative Sciences, Surgery, Umeå University, Umeå, Sweden; Department of Public Health and Clinical Medicine, Umeå University, Umeå, Sweden; Department of Surgical and Perioperative Sciences, Surgery, Umeå University, Umeå, Sweden; Department of Surgery, Faculty of Medicine and Health, Örebro University, Örebro, Sweden; Department of Radiation Sciences, Umeå University, Umeå, Sweden

## Abstract

**Background:**

Electrolyte disturbances and dehydration are common after anterior resection for rectal cancer with a defunctioning loop ileostomy. High-quality population-based studies on the impact of a defunctioning loop ileostomy on renal failure are lacking.

**Methods:**

This was a nationwide observational study, based on the Swedish Colorectal Cancer Registry of patients undergoing anterior resection for rectal cancer between 2008 and 2016, with follow-up until 2017. Patients with severe co-morbidity, with age greater than 80 years, and with pre-existing renal failure were excluded. Loop ileostomy at index surgery constituted exposure, while a diagnosis of renal failure was the outcome. Acute and chronic events were analysed separately. Inverse probability weighting with adjustment for confounding derived from a causal diagram was employed. Hazards ratios (HRs) with 95 per cent c.i. are reported.

**Results:**

A total of 5355 patients were eligible for analysis. At 5-year follow-up, all renal failure events (acute and chronic) were 7.2 per cent and 3.3 per cent in the defunctioning stoma and no stoma groups respectively. In the weighted analysis, a HR of 11.59 (95 per cent c.i. 5.68 to 23.65) for renal failure in ostomates was detected at 1 year, with the largest effect from acute renal failure (HR 24.04 (95 per cent c.i. 8.38 to 68.93)). Later follow-up demonstrated a similar pattern, but with smaller effect sizes.

**Conclusion:**

Patients having a loop ileostomy in combination with anterior resection for rectal cancer are more likely to have renal failure, especially early after surgery. Strategies are needed, such as careful fluid management protocols, and further research into alternative stoma types or reduction in stoma formation.

## Teaser

This nationwide study of 5355 patients undergoing anterior resection for rectal cancer evaluated the use of defunctioning loop ileostomies in relation to subsequent renal failure events. A substantial increase in acute renal failure was noted in the ileostomy group, especially in the first postoperative year. Countermeasures and alternatives for ileostomies should be considered.

## Introduction

Anterior resection is the most common operative strategy for treating high- and mid-rectal cancer. A defunctioning stoma is fashioned in some patients to reduce morbidity and mortality^1^ rates that may result as a consequence of an anastomotic leakage in higher-risk anastomoses^2^. A loop ileostomy is more often chosen, compared with loop colostomy, as an ileostomy is considered to be less challenging to construct and reverse later. Ileostomies are less likely to prolapse, and have lower rates of wound infection and incisional hernia after reversal^3^.

However, loop ileostomies have their own complication profile including high stoma output^3^ and readmission rates due to dehydration of 6 per cent within 30 days^4^. Some of these patients can develop acute renal failure^5^, which may progress to chronic kidney disease^6^. There are single-centre cohort data on patients with rectal cancer showing that defunctioning loop ileostomies are associated with renal impairment, even after stoma closure^7^, and database studies claim a two- to three-fold increased risk of acute renal insufficiency compared with patients without a stoma^8,9^. A large population-based study on all types of ileostomy creation found a four-fold increased risk of acute kidney injury in ostomates within 3 months of surgery, and this risk progressed to chronic disease within a year, notwithstanding stoma reversal^10^.

While these studies suggest a causative effect of loop ileostomy formation on renal impairment, selection bias and residual confounding is an issue in some studies, while the target population of rectal cancer has not been explicitly studied in others. To evaluate this in a true population-based setting, a nationwide study using registry-based data was conducted, taking into account known confounding and time-to-event data. The main hypothesis was that formation of a defunctioning loop ileostomy causes postoperative renal failure after anterior resection for rectal cancer, while the secondary hypothesis was that early stoma reversal mitigates this effect.

## Methods

### Ethics approval statement

The study was approved by the Regional Board of the Ethics Committee in Umeå, Sweden (DNR: 2011-234-31M, 2015-6-32, 2015-122-31, 2018-81-32).

### Checklist for the reporting of observational studies

This article was written in accordance with the STROBE checklist for the reporting of observational studies^11^.

### Data source

A nationwide registry study was conducted based on the Swedish Colorectal Cancer Registry (SCRCR), which contains nearly all Swedish patients diagnosed with colorectal cancer. For rectal cancer, the registration started in 1995 and the mean completeness in 2008–2015 was 98.8 per cent^12^. In the SCRCR, a rectal cancer is defined as an adenocarcinoma with its height 15 cm from the anal verge or less as measured with rigid sigmoidoscopy. Data are prospectively recorded during treatment and follow-up, while validation studies using re-abstraction have shown a median accuracy over 90 per cent for the registry variables^12^. Patient and tumour characteristics such as age, sex, ASA fitness grade, tumour location, and tumour stage are reported in detail, as well as preoperative treatment and perioperative data including type of surgery and postoperative complications, but also long-term follow-up such as recurrence and survival. To complement the data derived from the SCRCR, the National Patient Registry was used to include codes for different diagnoses and operations, as well as corresponding dates. Data on inpatient care have been collected since 1964 (with nationwide coverage since 1987) and outpatient data since 2001, with a national coverage of more than 99 per cent^13^. This registry has also been validated several times, with positive predictive values for the registered diagnosis codes ranging from 85 to 95 per cent^13^.

### Study design

Patients treated with an anterior resection for rectal cancer in Sweden between 1 January 2008 and 31 December 2016 were identified using the SCRCR. These patients were subsequently linked to the National Patient Registry, using the national personal identification number^14^, covering diagnoses, operations, and corresponding dates from 1 January 2007 to 31 December 2017, constituting last date of follow-up. To emulate a trial context, exclusion criteria comprised a diagnosis of chronic renal failure before index surgery, ASA fitness grade IV, and age over 80 years. Moreover, patients with a loop colostomy (ICD codes JFF20, JFF23, JFF26, JFF30, or JFF31) were also excluded from analysis.

While the SCRCR was used to extract surgery-related and demographic variables, the National Patient Registry was used to collect data on co-morbidities. These included hypertension, preoperative chronic renal failure, diabetes, and chronic obstructive pulmonary disease (*Table S1*).

### Exposure

The SCRCR was used to determine whether patients had received a defunctioning stoma, while surgical codes from the National Patient Registry were extracted to establish stoma type. Concerning the exposure, these were categorized into ileostomy or unspecified.

### Outcome

The National Patient Registry was subsequently used to extract information regarding existence of renal failure and time of diagnosis. The ICD code N17 was used for acute renal failure, while the codes N18, N19, N99, I12.0, I13.0, and I13.2 were used for chronic renal failure. The main outcome in this study was any renal failure (both acute and chronic), while secondary outcomes included acute or chronic renal failure. The outcomes were recorded at 1, 3 and 5 years after index surgery.

### Statistical analysis

Frequency tables concerning patient characteristics, tumour stage, and operative details were constructed. Continuous variables are described using the median along with the interquartile range (i.q.r.).

**Fig. 1:**
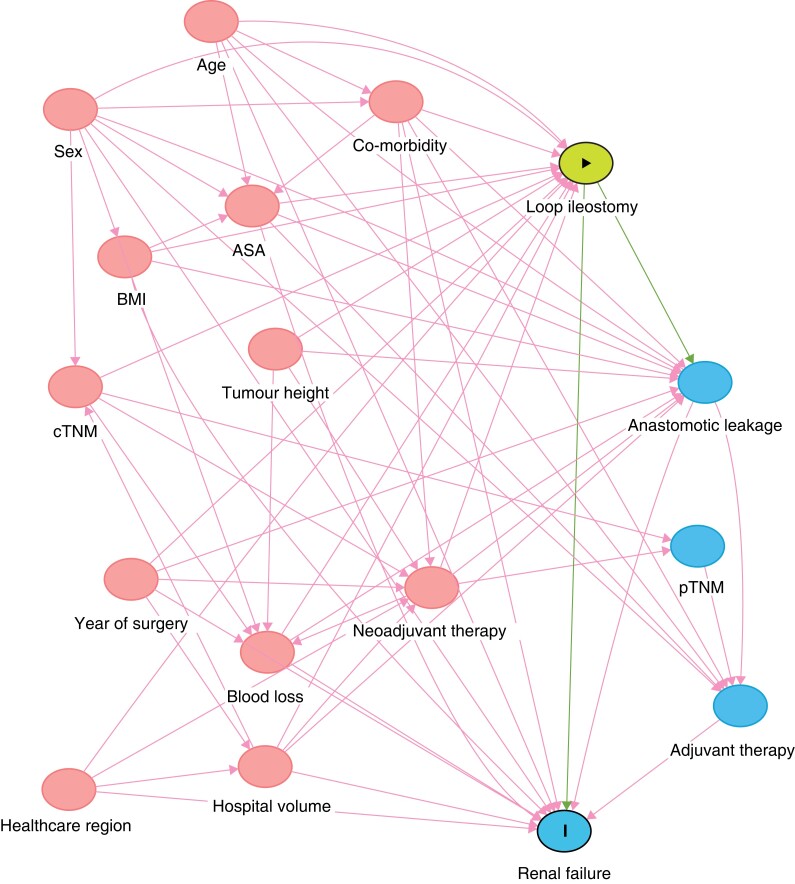
Directed acyclic graph, depicting the proposed relationships between exposure, outcome, and other variables pertaining to the research question Red circles indicate ancestors of both outcome and exposure, necessary to adjust to eliminate confounding. cTNM, clinical tumour node metastasis staging system; pTNM, pathological tumour node metastasis staging system.

A directed acyclic graph was constructed to visualize the authors’ understanding of the causal pathways involved in the potential effects conveyed by stoma formation^15^. With this diagram in mind, confounders were selected to estimate the total effect of a defunctioning loop ileostomy on renal failure (*Fig. 1*). In the main analyses, a defunctioning stoma was considered the exposure for renal failure (all; chronic; acute). ASA fitness grade (I, II, or III), age (continuous; years), healthcare region, hospital volume (continuous; caseload per year), perioperative bleeding (continuous; ml), neoadjuvant treatment (none, radiotherapy, or chemoradiotherapy), sex (male or female), clinical tumour (T1–2, T3, T4, or undefined), node (N0, N1–2, or undefined) and metastasis (M0, M1, or undefined) categories, year of operation (categorical), co-morbidities (presence of each: yes or no), tumour height (continuous; cm), and BMI (continuous; kg/m^2^) were considered to be confounding variables.

Propensity score weighting was used to adjust for potential confounding, aiming to emulate a randomized trial setting, where all analysed patients would have the same likelihood of receiving a defunctioning stoma. Propensity scores were calculated using a logistic regression model and propensity score weights suitable for estimating the average treatment effect were calculated. Absolute mean standardized differences below 0.25 and 0.10 were considered as indicative of an acceptable and a good balance after weighting respectively.

The associations of defunctioning stoma with renal failure were visualized using Kaplan–Meier (K–M) curves, and differences were evaluated using the log rank test. Treating death as censored (cause-specific K–M) instead of considering it a competing risk (competing risk K–M) tends to bias the survival curves; therefore both types of curves are shown in the K–M figures. Risk differences were also calculated based on the competing risk K–M curves. Cox proportional-hazards regression was performed using the propensity score weights, including defunctioning stoma as the only covariate. In addition, as a comparison, univariable regression analyses were performed on the unweighted data.

Using the subsample of patients with defunctioning stoma, subsequent stoma reversal, and follow-up time longer than 90 days, the effect of a stoma reversal within 90 days on renal failure was assessed in a separate analysis. Here, patients with renal failure or mortality occurring within 90 days were excluded from analysis, thus retaining only living patients without any renal failure at analysis start. The confounder set included ASA fitness grade, age, healthcare region, co-morbidities, anastomotic leakage, year of operation, and pathological tumour stage. Propensity scores were calculated using a logistic regression model, while absolute mean standardized differences were again derived using the same threshold as above.

Finally, the main analysis and the early stoma reversal analysis were repeated when excluding stoma types of uncertain type, to ascertain whether the assumption was correct that unspecified stoma type in reality denoted a loop ileostomy.

Throughout, a complete case analysis was employed. All analyses were performed using the statistical software R 4.1.3 (R: A Language and Environment for Statistical Computing. R Foundation for Statistical Computing, Vienna, Austria).

## Results

### Patients

**Fig. 2:**
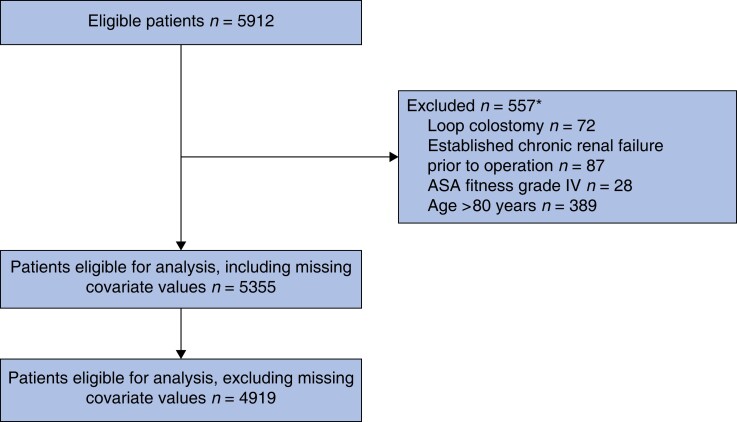
Study flow chart, analysing the effect of defunctioning loop ileostomy on renal failure Covariates with missing values were: ASA fitness grade, BMI, perioperative bleeding, clinical tumour category, clinical node category, clinical metastasis category, and tumour height. *The total number of patients excluded does not add up, as some patients fulfilled more than one exclusion criterion.

In total, 5912 patients identified in the SCRCR had an anterior resection for rectal cancer during 2008–2016. Of these, 557 patients were excluded due to loop colostomy use, old age (greater than 80 years), severe co-morbidity (ASA fitness grade greater than or equal to IV), and a prior diagnosis of chronic kidney disease. Notably, defunctioning stomas were used in 81.5 per cent (4364) of the cases; of these, 77.8 per cent (3395) were coded as loop ileostomies, while 22.2 per cent (969) were of an unspecified type. Some 5355 patients were eligible for analysis, including those with missing co-variates and 4919 with complete data for all covariates (*Fig. 2*).

Clinical and demographic data of the cohort are presented in *Table 1*. It is notable that patients with a defunctioning loop ileostomy, in comparison with those without, were more often men, were slightly younger, had a lower tumour height with a more advanced tumour, and hence had more often received neoadjuvant therapy. However, co-morbidity measured by ASA fitness grade, as well as by diagnostic codes, was similar between groups. About half of the potential confounders exhibited some missing data, with at most 3.7 per cent incomplete data for these variables.

**Table 1:** Clinical and demographic data for 5355 patients (patients with missing covariate values included)

Variables	No defunctioning stoma (*n* = 991)	Defunctioning stoma (*n* = 4364)	Overall (*n* = 5355)
**Sex**			
Male	504 (50.9)	2624 (60.1)	3128 (58.4)
Female	487 (49.1)	1740 (39.9)	2227 (41.6)
**Age (years), median (i.q.r.)**	68 (60–73)	66 (59–72)	66 (59–72)
**BMI (kg/m^2^), median (i.q.r.)**	25.5 (23.2–28.7)	25.5 (23.3–28.1)	25.5 (23.3–28.1)
Missing	51 (5.1)	149 (3.4)	200 (3.7)
**ASA fitness grade**			
I	260 (26.2)	1134 (26.0)	1394 (26.0)
II	565 (57.0)	2561 (58.7)	3126 (58.4)
III	158 (15.9)	625 (14.3)	783 (14.6)
Missing	8 (0.8)	44 (1.0)	52 (1.0)
**Clinical tumour category**			
cT1–cT2	333 (33.6)	1101 (25.2)	1434 (26.8)
cT3	437 (44.1)	2494 (57.1)	2931 (54.7)
cT4	65 (6.6)	535 (12.3)	600 (11.2)
cTx	113 (11.4)	206 (4.7)	319 (6.0)
Missing	43 (4.3)	28 (0.6)	71 (1.3)
**Clinical node category**			
cN0	525 (53.0)	1766 (40.5)	2291 (42.8)
cN1–cN2	328 (33.1)	2285 (52.4)	2613 (48.8)
cNx	127 (12.8)	306 (7.0)	433 (8.1)
Missing	11 (1.1)	7 (0.2)	18 (0.3)
**Clinical metastasis category**			
cM0	910 (91.8)	4043 (92.6)	4953 (92.5)
cM1	56 (5.7)	278 (6.4)	334 (6.2)
cMx	20 (2.0)	39 (0.9)	59 (1.1)
Missing	5 (0.5)	4 (0.1)	9 (0.2)
**Neoadjuvant therapy**			
None	692 (69.8)	1249 (28.6)	1941 (36.2)
Radiotherapy	230 (23.2)	2116 (48.5)	2346 (43.8)
Chemoradiotherapy	69 (7.0)	999 (22.9)	1068 (19.9)
**Hypertension**	340 (34.3)	1430 (32.8)	1770 (33.1)
**Cardiovascular disease**	82 (8.3)	364 (8.3)	446 (8.3)
**Heart failure**	19 (1.9)	108 (2.5)	127 (2.4)
**Diabetes**	95 (9.6)	448 (10.3)	543 (10.1)
**Chronic obstructive pulmonary disease**	30 (3.0)	110 (2.5)	140 (2.6)
**Surgical technique**			
Open	726 (73.3)	3576 (81.9)	4302 (80.3)
Laparoscopy	214 (21.6)	610 (14.0)	824 (15.4)
Converted to open	42 (4.2)	152 (3.5)	194 (3.6)
Missing	9 (0.9)	26 (0.6)	35 (0.7)
**Anastomotic leakage**	90 (9.1)	391 (9.0)	481 (9.0)
**Healthcare region**			
Stockholm-Gotland	135 (13.6)	955 (21.9)	1090 (20.4)
Mid-Sweden	236 (23.8)	1041 (23.9)	1277 (23.8)
Southeastern	161 (16.2)	427 (9.8)	588 (11.0)
Southern	146 (14.7)	855 (19.6)	1001 (18.7)
Western	247 (24.9)	779 (17.9)	1026 (19.2)
Northern	66 (6.7)	307 (7.0)	373 (7.0)
**Stage (pathological)**			
I	302 (30.5)	1278 (29.3)	1580 (29.5)
II	270 (27.2)	1169 (26.8)	1439 (26.9)
III	336 (33.9)	1553 (35.6)	1889 (35.3)
IV	63 (6.4)	295 (6.8)	358 (6.7)
Missing	20 (2.0)	69 (1.6)	89 (1.7)
**Perioperative bleeding (ml), median (i.q.r.)**	200 (75–400)	350 (150–600)	300 (100–600)
Missing	35 (3.5)	100 (2.3)	135 (2.5)
**Tumour height (cm), median (i.q.r.)**	13 (12–14)	10 (8–12)	10 (8–13)
Missing	15 (1.5)	19 (0.4)	34 (0.6)
**Hospital volume (operations/year), median (i.q.r.)**	16.8 (12.7–23.6)	18.7 (13.8–24.9)	18.4 (13.8–24.9)
**Operation year, median (i.q.r.)**	2012 (2009–2014)	2012 (2010–2014)	2012 (2010–2014)

Values are *n* (%) unless otherwise indicated.

### Stoma formation and renal failure with background mortality

In the entire cohort, any renal failure was diagnosed at a median of 338 (i.q.r. 46–1404) days, while acute and chronic renal failure were diagnosed at a median of 93 (i.q.r. 30–627) days and 1208 (i.q.r. 442–1977) days after surgery respectively. Divided by different intervals of follow-up, patients with a defunctioning stoma displayed consistently higher rates of renal failure than those without at 1, 3, or 5 years after surgery, while mortality was similar (*Table 2*).

**Table 2:** Frequency of events (any renal failure and death) during 1-, 3-, and 5-year follow-up among the 4919 patients

Event type	No defunctioning stoma (*n* = 860)	Defunctioning stoma (*n* = 4059)
**1-year follow-up**		
Renal failure	9 (1.0)	191 (4.7)
Death	15 (1.7)	137 (3.4)
**3-year follow-up**		
Renal failure	17 (2.0)	241 (5.9)
Death	76 (8.8)	414 (10.2)
**5-year follow-up**		
Renal failure	28 (3.3)	291 (7.2)
Death	120 (14.0)	605 (14.9)

Values are *n* (%).

### Impact of stoma formation on renal failure

**Fig. 3:**
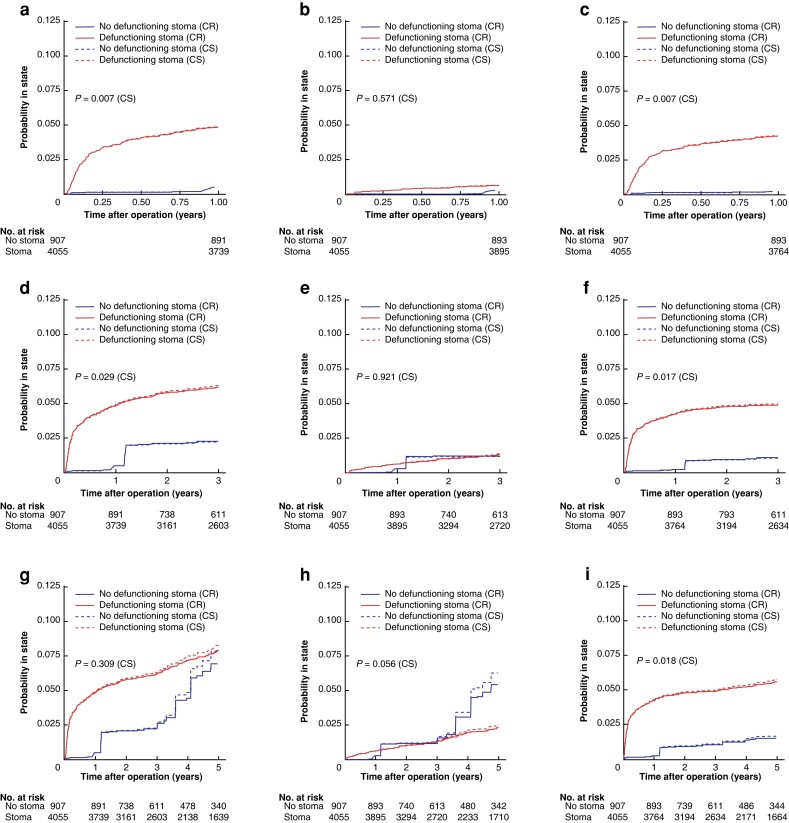
Kaplan–Meier curves on propensity score-weighted data, by presence of defunctioning stoma, on renal failure when death is censored (CS; dashed curves) and renal failure when death is considered a competing risk (CR; solid curves) First row, 1-year follow-up: **a**, any renal failure; **b**, chronic renal failure; and **c**, acute renal failure. Second row, 3-year follow-up: **d**, any renal failure; **e**, chronic renal failure; and **f**, acute renal failure. Third row, 5-year follow-up: **g**, any renal failure; **h**, chronic renal failure; and **i**, acute renal failure. Log rank tests for curves with death censored.

The balance achieved before and after propensity-scored weighting can be seen in *Fig. S1*, where the absolute mean differences were below 0.11 for all covariates after weighting. Time to renal failure in the weighted cohort is visualized in *Fig. 3* (unweighted curves can be found in *Fig. S2*). Here, both competing risk and cause-specific K–M curves are presented, although these approaches produced similar results. The weighted K–M curves show an apparent strong early effect within the first postoperative year in patients with a defunctioning stoma, after which the K–M curves seem to be approximately parallel up to 5 years. Most of the effect shown is consistent with an impact from acute renal failure, while chronic renal failure events were similar between groups. The K–M curves incorporating acute renal failure all show statistically significant differences (log rank *P* < 0.050).

The corresponding risk differences and hazard ratios (HRs) for time to renal failure by defunctioning stoma are presented in *Table 3*. Notably, while the main outcome ‘any renal failure’ (within the first postoperative year) was affected by having a defunctioning stoma with an HR of 11.59 (95 per cent c.i. 5.68 to 23.65) in the weighted analysis, this seemed to be driven by acute renal failure (HR 24.04 (95 per cent c.i. 8.38 to 68.93)). The point estimates were attenuated with longer duration of follow-up, but the same pattern persisted. In the unweighted analysis, point estimates were generally lower, but the effects were consistent with the adjusted model (*Table 3*).

**Table 3:** Risk differences and hazards ratios, with 95 per cent confidence intervals, with defunctioning stoma as exposure and renal failure as outcome; analyses are based on the complete case data with *n* = 4919

Outcome	1-year		3-year		5-year	
	RD (95% c.i.)	HR (95% c.i.)	RD (95% c.i.)	HR (95% c.i.)	RD (95% c.i.)	HR (95% c.i.)
**Weighted data**						
Any renal failure	0.04 (0.04 to 0.05)	11.59 (5.68 to 23.65)	0.05 (0.03 to 0.06)	4.16 (1.82 to 9.53)	0.03 (0.00 to 0.06)	1.98 (1.11 to 1.35)
Chronic renal failure	0.00 (0.00 to 0.01)	2.47 (0.90 to 6.81)	0.01 (0.00 to 0.01)	1.76 (0.54 to 5.73)	−0.01 (−0.04 to 0.01)	0.73 (0.34 to 1.53)
Acute renal failure	0.04 (0.03 to 0.05)	24.04 (8.38 to 68.93)	0.04 (0.03 to 0.05)	6.31 (1.96 to 20.30)	0.04 (0.03 to 0.06)	5.16 (2.06 to 12.93)
						
**Unweighted data**						
Any renal failure	0.04 (0.03 to 0.05)	4.63 (2.38 to 9.01)	0.04 (0.03 to 0.05)	3.12 (1.91 to 5.09)	0.04 (0.02 to 0.06)	2.32 (1.58 to 3.40)
Chronic renal failure	0.00 (−0.01 to 0.01)	1.06 (0.41 to 2.78)	0.00 (0.00 to 0.01)	1.43 (0.68 to 3.02)	0.00 (−0.01 to 0.02)	1.22 (0.70 to 2.12)
Acute renal failure	0.04 (0.03 to 0.04)	9.08 (3.37 to 24.46)	0.04 (0.03 to 0.05)	4.62 (2.37 to 8.99)	0.04 (0.03 to 0.05)	3.58 (2.05 to 6.25)

RD, risk difference; HR, hazards ratio.

### Effect of stoma reversal

**Fig. 4:**
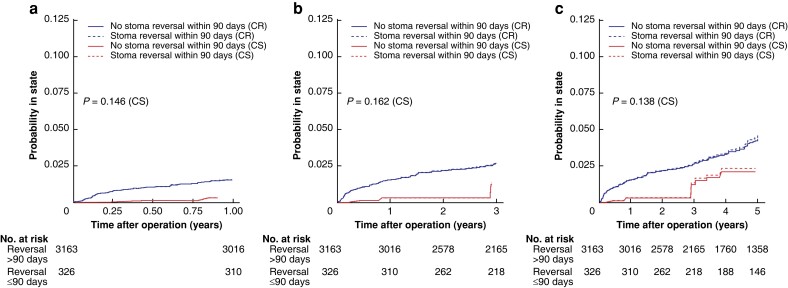
Kaplan–Meier curves on propensity score-weighted data consisting only of patients with defunctioning stoma and subsequent stoma reversal, by presence of stoma reversal within 90 days, on any renal failure when death is censored (CS; dashed curves) and any renal failure when death is considered a competing risk (CR; solid curves) **a** One-year follow-up. **b** Three-year follow-up. **c** Five-year follow-up. Log rank tests for curves with death censored.

From the 3599 patients in the original analysis cohort, with a defunctioning stoma in place after index surgery and subsequent stoma reversal, 105 patients were excluded as mortality or renal failure had occurred within 90 postoperative days. The remaining 3494 patients with follow-up beyond 90 days are described in *Table S2*. Of note, any renal failure was less prevalent in the group with early stoma reversal (within 90 days) (2.1 per cent), in comparison with the group with a later reversal (3.6 per cent) (5-year follow-up; see *Table S3*). After propensity-scored weighting, the balance achieved was good, with absolute mean differences below 0.10 for all covariates (*Fig. S3*). Time to all renal failure in the weighted data is described in *Fig. 4*, with competing risk and cause-specific curves (unweighted data are described in *Fig. S4*). The K–M curves display early separation, with few events in the first postoperative interval for the early reversal group (log rank *P* values between 0.14 and 0.16). The corresponding risk differences and HRs are shown in *Table 4*. The risk of renal failure was substantially reduced within 1 year after surgery in the early stoma reversal group with an HR of 0.19 (0.95 per cent c.i. 0.05 to 0.83), where later follow-up times displayed attenuated reductions, though statistically non-significant (*Table 4*).

**Table 4:** Risk differences and hazards ratios, with 95 per cent confidence intervals, with early stoma reversal (less than or equal to 90 days) as exposure and any renal failure as outcome; analyses are based on the complete case data with *n* = 3494 (early stoma reversal analysis)

Outcome	1-year		3-year		5-year	
	RD (95% c.i.)	HR (95% c.i.)	RD (95% c.i.)	HR (95% c.i.)	RD (95% c.i.)	HR (95% c.i.)
**Weighted data**						
Any renal failure	−0.01 (−0.02 to −0.01)	0.19 (0.05 to 0.83)	−0.01 (−0.03 to 0.00)	0.40 (0.12 to 1.34)	−0.02 (−0.04 to 0.00)	0.46 (0.19 to 1.12)
**Unweighted data**						
Any renal failure	−0.01 (−0.02 to 0.00)	0.39 (0.10 to 1.60)	−0.01 (−0.03 to 0.00)	0.47 (0.17 to 1.27)	−0.02 (−0.04 to 0.00)	0.57 (0.27 to 1.23)

RD, risk difference; HR, hazards ratio.

### Sensitivity analysis

After excluding patients with an unspecified stoma type, results were similar concerning main analyses (*Table S4*), as well as the early stoma reversal analysis (*Table S5*).

## Discussion

In this nationwide registry-based study, a defunctioning loop ileostomy in conjunction with anterior resection for rectal cancer surgery was strongly associated with renal failure. This effect was almost completely due to acute renal failure, especially pronounced in the first postoperative year. The results from the secondary analysis of early stoma reversal suggested causality, as earlier reversals seem to mitigate the effect of loop ileostomy on early renal failure. Due to the registry-based nature of the study, not all defunctioning stomas were possible to characterize, but excluding the unspecified stoma patients did not alter the results, corroborating the assumption that the vast majority of such stomas were indeed loop ileostomies.

Consistent with physiology and clinical experience, meta-analysis data from trials show that stoma output is increased with a loop ileostomy compared with a loop colostomy^3^. While certainly plausible, trial data are not available evaluating any reduction of renal function; nevertheless, there are numerous observational studies. Fielding *et al*.^7^ conducted a single-centre study of 1213 patients with rectal cancer, where a low anterior resection group with loop ileostomy was compared with high anterior resection without such a stoma and abdominoperineal excision with a colostomy. The mean estimated glomerular filtration rate (eGFR) was lower in the ileostomy group, and the rate of moderate to severe chronic kidney disease increased from 13 per cent at index surgery to 23 per cent at time of stoma closure, taking place at a median of 189 days after surgery; notably, this did not improve after stoma closure^7^. While this study is more detailed concerning the outcome, reflected in higher rates of renal impairment in comparison with the present study, it is not population-based. A database study including 15 075 anterior resections for rectal cancer found that patients with defunctioning stomas had a higher likelihood of readmission for dehydration, renal failure, and progressive renal insufficiency compared with non-diverted patients. Interestingly, the increased risks were similar regardless of ileostomy or colostomy use^9^. However, the statistical methodology was somewhat unclear regarding adjustment for confounding, as the included covariates were not explicitly stated and the most important confounder, tumour height, was not available. Smith *et al*.^10^ performed a population-based study of 19 889 patients with detailed serum creatinine data, comprising an ileostomy group (new stoma with or without bowel resection) and a control group of patients with bowel resection, but without ileostomy. Community-onset (excluding inpatient) acute kidney injury within 3 months after surgery was 15 *versus* 5 per cent in the stoma and no stoma groups respectively. New-onset chronic kidney disease occurred in 6 *versus* 2 per cent within the year. Acute kidney injury events predisposed to the development of chronic disease, while stoma reversal within a year halted this progression, although only completely in those without a previous acute kidney injury. This study demonstrates lower rates of both acute and chronic renal failure than the previous report as a consequence of the registry-based ascertainment; however, Smith *et al*.^10^ did not use time-to-event data or evaluate anterior resections exclusively. The statistical methodology was not aimed at studying causal effects, evidenced by the use of mediators such as in-patient acute kidney injury in their regression models. Although the results are similar to this study, their findings are more difficult to interpret. This study could not establish that chronic renal failure was increased by the use of a defunctioning loop ileostomy, possibly due to low event rates constituting a type II error, but also by probable misclassification between acute and chronic events in the registry. The finding of the impact of early stoma reversal (less than or equal to 90 days after index surgery) on renal failure is contradicted by some smaller cohort studies, where renal impairment was detected even before stoma closure within 2–3 months of the index procedure^16,17^. A RCT on early closure (8–13 days) reported that high stoma output was more common in the delayed reversal group, though numbers were too small to draw any conclusion, while data on renal impairment were not recorded^18^. However, Yang *et al*.^19^ reported from their study of 320 patients with rectal cancer that, while ileostomy patients exhibited renal impairment (eGFR less than 60 ml/min/1.73 m^2^) at 3 months (6 per cent *versus* 1 per cent in controls), this impairment was partly resolved after stoma closure (taking place within 2 years of the index surgery). In summary, most evidence points to a real risk of causing renal damage with the use of a defunctioning loop ileostomy, while this could only partially be reduced by early reversal surgery.

Severe renal failure increases in-hospital mortality by six times in patients with colorectal cancer^20^. While renal failure-related mortalities with a defunctioning loop ileostomy are uncommon, frailer patients are at risk for persisting renal impairment^5^. The present study suggests a causal effect of loop ileostomy formation on the development of renal failure both acutely with the risk of progression to chronic kidney disease and other long-term effects such as cardiovascular mortality^21,22^. The results of the present study could motivate a more judicious approach to defunctioning stomas, as well as studies evaluating alternative measures such as loop colostomies. While defunctioning stomas seem to reduce at least symptomatic anastomotic leakage and the need for reoperation^1^, there are data suggesting that the long-term risk of a permanent stoma is increased^23^. In patients that have a loop ileostomy, increased vigilance for the risk of dehydration and renal failure is certainly warranted, including careful fluid management^24^.

The major strengths of the current study are the large sample size and the population-based nature, where even minor effects can be discerned and selection bias is alleviated, respectively. Modern statistical methods have been used within a causal framework, in particular reporting our assumptions of the putative relations between important variables using a directed acyclic graph. Utilizing strict inclusion and exclusion criteria, as well as propensity-score based weighting, enables greater confidence in conclusions, accepting the observational design^25^. There are limitations including residual confounding encountered in observational studies and misclassification error. The latter is pertinent to this study in particular and would constitute one of the major weaknesses, as there were no available laboratory (for example serum creatinine) or clinical (for example urine output) data to determine the renal failure diagnosis; ideally, such a diagnosis should be supported by strict parameters according to the Kidney Disease: Improving Global Outcomes (KDIGO) guidelines^26^. In the present study, the outcomes were derived entirely from registry data, relying on clinicians to state a correct diagnosis that is subsequently captured accurately by the National Patient Registry. This inevitably leads to misclassification, albeit in all likelihood non-differential in nature; judging from the comparably low proportions of renal failure in this study compared with others^27^, it is probable that the true rate of renal failure in this cohort is higher than the registered rate; the exclusion of older and more co-morbid patients certainly contributed to the low rates. However, such misclassification would likely lead to dilution of the association towards the null hypothesis, whereas this study still displayed clear effects of defunctioning stoma use on renal failure.

## Supplementary Material

zrad010_Supplementary_Data

## Data Availability

Upon reasonable request, data can be shared, subject to approval from the steering committee of the Swedish Colorectal Cancer Registry.
