# Corrigendum to: Defunctioning loop ileostomy in anterior resection for rectal cancer and subsequent renal failure: nationwide population-based study

**DOI:** 10.1093/bjsopen/zrad071

**Published:** 2023-06-23

This is a Corrigendum to: Martin Rutegård and others, Defunctioning loop ileostomy in anterior resection for rectal cancer and subsequent renal failure: nationwide population-based study, *BJS Open*, Volume 7, Issue 3, June 2023, zrad010, https://doi.org/10.1093/bjsopen/zrad010

In the originally published version of this manuscript, there were errors in Figure 3. Beneath each ‘No. at risk’ the labels for numbers were transposed and these should read in order:

“No stoma

Stoma” instead of:

“Stoma

No stoma”.

There were errors in Figure 4. Beneath each ‘No. at risk’, the labels on panels a, b and c for the figures were errored. These should all read:

“Reversal >90 days

Reversal ≤90 days” instead of:

“Stoma

No stoma”.

These errors have been corrected in the article.

