# Supplementary material for: Defunctioning loop ileostomy in anterior resection for rectal cancer and subsequent renal failure: nationwide population-based study
Source: BJS Open. 2023 May 10;7(3):zrad010. doi: 10.1093/bjsopen/zrad010 (PMC10170252; doi:10.1093/bjsopen/zrad010)
Supplement: zrad010_Supplementary_Data [file zrad010_supplementary_data.docx]

# Defunctioning loop ileostomy in anterior resection for rectal cancer and subsequent renal failure: nationwide population-based study

### Authors:

Martin Rutegård, MD, PhD (1,2), Jenny Häggström, PhD (3), Erik Back, MD (1), Klas Holmgren, MD, PhD (1), Jonas Wixner MD, PhD (4), Jörgen Rutegård MD, FRCS, PhD (1), Peter Matthiessen, MD, PhD (5), Olle Sjöström, MD, PhD (6).

### Affiliations:

1) Department of Surgical and Perioperative Sciences, Surgery, Umeå University, Umeå, Sweden.

2) Wallenberg Centre for Molecular Medicine, Umeå University, Umeå, Sweden.

3) Department of Statistics, Umeå School of Business, Economics and Statistics, Umeå University, Umeå, Sweden.

4) Department of Public Health and Clinical Medicine, Umeå University, Umeå, Sweden.

5) Department of Surgery, Faculty of Medicine and Health, Örebro University, Örebro, Sweden.

6) Department of Radiation Sciences, Umeå University, Umeå, Sweden.

### Corresponding author:

Martin Rutegård ([martin.rutegard@umu.se](mailto:martin.rutegard@umu.se))

Department of Surgical and Perioperative Sciences, Surgery, Umeå University

SE-901 85 Umeå, Sweden

ORCID ID 0000-0002-0974-6373

Twitter: @martin_rutegard

### Supplementary Materials - Index

| **Supplementary Figures and Tables** |  |
| --- | --- |
| Table S1 | *pag. 2* |
| Table S2 | *pag. 2-3* |
| Table S3 | *pag. 4* |
| Table S4 | *pag. 4* |
| Table S5 | *pag. 4* |
| Figure S1 | *pag. 5* |
| Figure S2 | *pag. 6* |
| Figure S3 | *pag. 7* |
| Figure S4 | *pag. 7* |
|  |  |

**Table S1.** ICD codes used to characterise comorbidity.

| Comorbidity | ICD code |
| --- | --- |
| Hypertension | I10, I11.9, I12.9, I13.9, I15 |
| Cardiovascular disease | I20, I25 (except I25.3, I25.4), I73.9 |
| Heart failure | I11.0, I13.0, I13.2, I50 |
| Chronic renal failure | I12.0, I13.1, I13.2, N18, N19, N99 |
| Diabetes | E10, E11, E12, E13, E14 |
| Chronic obstructive pulmonary disease | J44 |

**Table S2.** Clinical and demographic data for *n* = 3494 patients with defunctioning stoma, subsequent stoma reversal and follow-up time longer 90 days (patients with missing values on ASA fitness grade, and pathological tumour stage excluded)

|  | **No stoma reversal within 90 days**  **(*n* = 3163)** | **Stoma reversal within 90 days**  **(*n* = 331)** | **Overall**  **(*n* = 3494)** |
| --- | --- | --- | --- |
| **Categorical variables** | ***n* (%)** | ***n* (%)** | ***n* (%)** |
| **Sex** |  |  |  |
| Male | 1909 (60.4%) | 174 (52.6%) | 2083 (59.6%) |
| Female | 1254 (39.6%) | 157 (47.4%) | 1411 (40.4%) |
| **Age (years)** |  |  |  |
| Median (IQR) | 66 (59; 71) | 66 (58; 72) | 66 (59; 71) |
| **Body mass index** |  |  |  |
| Median (IQR) | 25.6 (23.4; 28.1) | 25.0 (22.7; 27.5) | 25.6 (23.3; 28.1) |
| **ASA fitness grade** |  |  |  |
| I | 874 (27.6%) | 98 (29.6%) | 972 (27.8%) |
| II | 1869 (59.1%) | 202 (61.0%) | 2071 (59.3%) |
| III | 420 (13.3%) | 31 (9.4%) | 451 (12.9%) |
| **Clinical tumour category** |  |  |  |
| cT1-cT2 | 836 (26.4%) | 90 (27.2%) | 926 (26.5%) |
| cT3 | 1800 (56.9%) | 199 (60.1%) | 1999 (57.2%) |
| cT4 | 370 (11.7%) | 18 (5.4%) | 388 (11.1%) |
| cTx | 137 (4.3%) | 22 (6.6%) | 159 (4.6%) |
| **Clinical node category** |  |  |  |
| cN0 | 1303 (41.2%) | 151 (45.6%) | 1454 (41.6%) |
| cN1-cN2 | 1656 (52.4%) | 151 (45.6%) | 1807 (51.7%) |
| cNx | 200 (6.3%) | 29 (8.8%) | 229 (6.6%) |
| **Clinical metastasis category** |  |  |  |
| cM0 | 2977 (94.1%) | 317 (95.8%) | 3294 (94.3%) |
| cM1 | 168 (5.3%) | 11 (3.3%) | 179 (5.1%) |
| cMx | 17 (0.5%) | 3 (0.9%) | 20 (0.6%) |
| **Neoadjuvant therapy** |  |  |  |
| None | 959 (30.3%) | 92 (27.8%) | 1051 (30.1%) |
| Radiotherapy | 1495 (47.3%) | 183 (55.3%) | 1678 (48.0%) |
| Chemoradiotherapy | 709 (22.4%) | 56 (16.9%) | 765 (21.9%) |
| **Hypertension** |  |  |  |
| No | 2169 (68.6%) | 257 (77.6%) | 2426 (69.4%) |
| Yes | 994 (31.4%) | 74 (22.4%) | 1068 (30.6%) |
| **Cardiovascular disease** |  |  |  |
| No | 2908 (91.9%) | 313 (94.6%) | 3221 (92.2%) |
| Yes | 255 (8.1%) | 18 (5.4%) | 273 (7.8%) |
| **Heart failure** |  |  |  |
| No | 3096 (97.9%) | 327 (98.8%) | 3423 (98.0%) |
| Yes | 67 (2.1%) | 4 (1.2%) | 71 (2.0%) |
| **Diabetes** |  |  |  |
| No | 2865 (90.6%) | 296 (89.4%) | 3161 (90.5%) |
| Yes | 298 (9.4%) | 35 (10.6%) | 333 (9.5%) |
| **Chronic obstructive**  **pulmonary disease** |  |  |  |
| No | 3091 (97.7%) | 326 (98.5%) | 3417 (97.8%) |
| Yes | 72 (2.3%) | 5 (1.5%) | 77 (2.2%) |
| **Surgical technique** |  |  |  |
| Open | 2601 (82.2%) | 249 (75.2%) | 2850 (81.6%) |
| Laparoscopy | 437 (13.8%) | 68 (20.5%) | 505 (14.5%) |
| Converted to open | 109 (3.4%) | 13 (3.9%) | 122 (3.5%) |
| **Anastomotic leakage** |  |  |  |
| No | 2933 (92.7%) | 314 (94.9%) | 3247 (92.9%) |
| Yes | 230 (7.3%) | 17 (5.1%) | 247 (7.1%) |
| **Healthcare region** |  |  |  |
| Stockholm-Gotland | 713 (22.5%) | 57 (17.2%) | 770 (22.0%) |
| Mid-Sweden | 779 (24.6%) | 61 (18.4%) | 840 (24.0%) |
| Southeastern | 328 (10.4%) | 14 (4.2%) | 342 (9.8%) |
| Southern | 594 (18.8%) | 87 (26.3%) | 681 (19.5%) |
| Western | 522 (16.5%) | 107 (32.3%) | 629 (18.0%) |
| Northern | 227 (7.2%) | 5 (1.5%) | 232 (6.6%) |
| **Stage (pathological)** |  |  |  |
| I | 954 (30.2%) | 133 (40.2%) | 1087 (31.1%) |
| I | 867 (27.4%) | 113 (34.1%) | 980 (28.0%) |
| III | 1173 (37.1%) | 72 (21.8%) | 1245 (35.6%) |
| IV | 169 (5.3%) | 13 (3.9%) | 182 (5.2%) |
| **Perioperative bleeding (ml)** |  |  |  |
| Median (IQR) | 350 (150; 600) | 300 (100; 500) | 350 (150; 600) |
| **Tumour height (cm)** |  |  |  |
| Median (IQR) | 10 (8; 12) | 10 (8; 12) | 10 (8; 12) |
| **Hospital volume** |  |  |  |
| Median (IQR) | 19.8 (14.2; 24.9) | 18.7 (13.8; 24.9) | 19.8 (14.1; 24.9) |
| **Operation year** |  |  |  |
| Median (IQR) | 2012 (2010; 2014) | 2012 (2010; 2014) | 2012 (2010; 2014) |

IQR = interquartile range; ASA = American Society of Anesthesiologists’

**Table S3.** Frequency of events (any renal failure and death) during 1-, 3-, and 5-year follow-up among the n = 3494 patients included in the early stoma reversal analysis.

| **Event type** | **No stoma reversal within 90 days**  **(n = 3163)** | **Stoma reversal within 90 days**  **(n = 331)** |
| --- | --- | --- |
|  | **n (%)** | **n (%)** |
|  | **1-year follow-up** | |
| **Any renal failure** | 49 (1.5%) | 2 (0.6%) |
| **Death** | 18 (0.6%) | 5 (1.5%) |
|  | **3-year follow-up** | |
| **Any renal failure** | 81 (2.6%) | 4 (1.2%) |
| **Death** | 171 (5.4%) | 23 (6.9%) |
|  | **5-year follow-up** | |
| **Any renal failure** | 113 (3.6%) | 7 (2.1%) |
| **Death** | 308 (9.7%) | 36 (10.9%) |

**Table S4.** Sensitivity analysis, patients with unspecified defunctioning stoma type excluded. Risk differences (RD) and hazard ratios (HR), with 95% confidence intervals (CIs), with defunctioning loop ileostomy as exposure and renal failure as outcome. Analyses are based on the complete cases data with n = 4014.

| **Outcome** | **1-year** | | **3-year** | | | | **5-year** | | | |
| --- | --- | --- | --- | --- | --- | --- | --- | --- | --- | --- |
|  | **RD (95% CI)** | **HR (95% CI)** | **RD (95% CI)** | | **HR (95% CI)** | | **RD (95% CI)** | | **HR (95% CI)** | |
| *Weighted data* |  | | | | | | | | | |
| **Any renal failure** | 0.05 (0.04, 0.06) | 12.01 (5.93, 24.33) | 0.05 (0.03, 0.06) | | 4.40 (1.96, 9.87) | | 0.03 (0.00, 0.06) | | 2.05 (1.15, 3.66) | |
| **Chronic renal failure** | 0.00 (0.00, 0.01) | 2.50 (0.89, 7.02) | 0.01 (0.00, 0.01) | | 1.82 (0.59, 5.59) | | -0.01 (-0.04, 0.01) | | 0.73 (0.34, 1.56) | |
| **Acute renal failure** | 0.04 (0.03, 0.05) | 24.75 (8.77, 69.84) | 0.04 (0.03, 0.05) | | 6.53 (2.05, 20.79) | | 0.04 (0.03, 0.06) | | 5.25 (2.12, 13.00) | |
|  |  |  |  | |  | |  | |  | |
| *Unweighted data* |  |  |  | |  | |  | |  | |
| **Any renal failure** | 0.04 (0.03, 0.05) | 5.00 (2.56, 9.76) | 0.04 (0.03, 0.06) | 3.27 (2.00, 5.36) | | 0.04 (0.03, 0.06) | | 2.40 (1.63, 3.54) | |  |
| **Chronic renal failure** | 0.00 (-0.01, 0.01) | 1.09 (0.41, 2.90) | 0.00 (0.00, 0.01) | 1.41 (0.66, 3.03) | | 0.00 (-0.01, 0.02) | | 1.23 (0.70, 2.16) | |  |
| **Acute renal failure** | 0.04 (0.03, 0.05) | 9.89 (3.67, 26.69) | 0.04 (0.03, 0.05) | 4.92 (2.52, 9.61) | | 0.04 (0.03, 0.05) | | 3.75 (2.14, 6.57) | |  |

**Table S5.** Sensitivity analysis, patients with unspecified defunctioning stoma type excluded. Risk differences (RD) and hazard ratios (HR), with 95% confidence intervals (CIs), with early stoma reversal (≤90 days) as exposure and any renal failure as outcome. Analyses are based on the complete cases data with n = 2767 (early stoma reversal analysis)

| **Outcome** | **1-year** | | **3-year** | | **5-year** | | |
| --- | --- | --- | --- | --- | --- | --- | --- |
|  | **RD (95% CI)** | **HR (95% CI)** | **RD (95% CI)** | **HR (95% CI)** | **RD (95% CI)** | **HR (95% CI)** | |
| *Weighted data* |  | | | | | |  |
| **Any renal failure** | -0.01 (-0.02, -0.01) | 0.21 (0.05, 0.88) | -0.02 (-0.03, 0.00) | 0.36 (0.09, 1.40) | -0.03 (-0.05, -0.01) | 0.40 (0.14, 1.09) | |
| *Unweighted data* |  | | | | | |  |
| **Any renal failure** | -0.01 (-0.02, 0.00) | 0.50 (0.12, 2.05) | -0.01 (-0.03, 0.00) | 0.45 (0.14, 1.43) | -0.02 (-0.04, 0.00) | 0.52 (0.21, 1.27) | |

**Figure S1.** Absolute mean differences of covariates in the data used for analysing the effect of defunctioning loop ileostomy on renal failure. ‘‘Unweighted’’ reflects differences before propensity score weighting; ‘‘Weighted’’ reflects differences after propensity score weighting. The dotted and dashed lines indicate absolute mean differences equal to 0.25 and 0.10, respectively. For continuous covariates the differences are standardized.

**Figure S2.** A–F, Kaplan-Meier curves on un-weighted data, by presence of defunctioning loop ileostomy, on renal failure when death is censored (CS; dashed curves) and renal failure when death is considered a competing risk (CR; solid curves). First row, 1-year follow-up: A, any renal failure; B, chronic renal failure; C, acute renal failure. Second row, 3-years follow-up: D, any renal failure; E, chronic renal failure; F, acute renal failure. Third row, 5-years follow-up: G, any renal failure; H, chronic renal failure; I, acute renal failure. Log-rank tests for curves with death censored.

**Figure S3.** Absolute mean differences of covariates in the data used for analysing the effect of stoma reversal within 90 days on renal failure. ‘‘Unweighted’’ reflects differences before propensity score weighting; ‘‘Weighted’’ reflects differences after propensity score weighting. The dotted and dashed lines indicate absolute mean differences equal to 0.25 and 0.10, respectively. For continuous covariates the differences are standardized.

**Figure S4.** Kaplan-Meier curves on unweighted data consisting only of patients with defunctioning stoma and subsequent stoma reversal, by presence of stoma reversal within 90 days, on any renal failure when death is censored (CS; dashed curves) and any renal failure when death is considered a competing risk (CR; solid curves). A, 1-year follow-up; B, 3-year follow-up; C, 5-year follow-up. Log-rank tests for curves with death censored.
